# The Parkinson's Disease Composite Scale Is Adequately Responsive to Acute Levodopa Challenge

**DOI:** 10.1155/2019/1412984

**Published:** 2019-09-10

**Authors:** Dávid Pintér, Pablo Martinez-Martin, József Janszky, Norbert Kovács

**Affiliations:** ^1^Doctoral School of Clinical Neuroscience, University of Pécs, Pécs, Hungary; ^2^National Center of Epidemiology, Carlos III Institute of Health, Madrid, Spain; ^3^Department of Neurology, Medical School, University of Pécs, Pécs, Hungary; ^4^MTA-PTE Clinical Neuroscience MR Research Group, Pécs, Hungary

## Abstract

**Background:**

The Parkinson's Disease Composite Scale (PDCS) is a recently developed easy-to-use tool enabling a timely but comprehensive assessment of Parkinson's disease (PD)-related symptoms. Although the PDCS has been extensively validated, its responsiveness to acute levodopa challenge has not been demonstrated yet.

**Objective:**

To investigate the correlation between changes in the motor examination part of the Movement Disorder Society-sponsored Unified Parkinson's Disease Rating Scale (MDS-UPDRS) and the PDCS motor scores during acute levodopa challenge and calculate a cutoff range on the PDCS indicating clinically relevant improvement.

**Methods:**

A consecutive series of 100 patients with parkinsonism were assessed using the motor examination sections of the MDS-UPDRS and the PDCS at least 12 hours after the last levodopa dose and after the administration of a single dose of a suprathreshold immediate formulation of levodopa/benserazide reaching the “best ON.” *Result*s. There was a high correlation between changes in the MDS-UPDRS and the PDCS motor scores (Spearman's rho = 0.73, *p* < 0.001). Receiver operating characteristic analysis revealed that a 14.6%–18.5% improvement in the PDCS motor scores corresponds to a 20–30% improvement in the MDS-UPDRS motor examination.

**Conclusions:**

The PDCS can reliably and adequately respond to an acute levodopa challenge. Any improvements in PDCS motor scores exceeding the 14.6–18.5% threshold could represent a clinically relevant response to levodopa.

## 1. Introduction

Parkinson's disease (PD) is associated with numerous and quite heterogeneous symptoms. Therefore, there is a high need from both clinical and research perspectives to comprehensively assess these problems. Although various tools, including the Movement Disorder Society-sponsored Unified Parkinson's Disease Rating Scale (MDS-UPDRS) [1], the Hoehn and Yahr Scale (HYS) [2], the Clinical Impression of Severity Index for Parkinson's Disease (CISI) [3], the Non-Motor Symptoms Scale (NMSS) [4], the Unified Dyskinesia Rating Scale [5], and the Montreal Cognitive Assessment (MoCA) [6], are available, these instruments still have some weaknesses. While the MDS-UPDRS measures the PD-related symptoms in a holistic approach, assessment of this scale is time-consuming. On the other hand, the use of the other aforementioned scales is somewhat faster and simpler; however, they focus on certain problems and consequently cannot provide a comprehensive picture. Moreover, the majority of the available assessment tools (e.g., MDS-UPDRS and MoCA) are copyrighted, which may limit their applicability. Because there was no single validated, reliable, highly responsive, and timely assessable tool, which can holistically measure the main motor and nonmotor symptoms of PD and suitable for both everyday clinical practice and research purposes, the European Parkinson's Disease Association sponsored the development of the Parkinson's Disease Composite Scale (PDCS).

Introduced in 2016 [7], the PDCS is a rater-based simple and relatively quick instrument measuring various aspects of PD including the severity of motor (6 items) and nonmotor symptoms (6 items), treatment-related complications (4 items), and PD-related disability (1 item). Motor symptoms are evaluated by the healthcare professional at the time of the visit, while nonmotor symptoms, treatment-related complications, and PD-related disability are rated based on the experience of the patient over the two weeks prior to the examination. Although each item can be rated as absent, mild, moderate, severe, or very severe, the scoring is asymmetric. Some clinically relevant problems (e.g., cognitive problems, postural instability, and falls) are scored from 0 to 7, while other less disabling problems have a range of 0–4. This unique scoring system enables the weighted measure of clinically disabling symptoms. Besides calculating the different domains, a total score can also be formulated.

The first validation study on the PDCS has found that this new scale seems to be feasible, acceptable, reproducible, valid, and precise [8]. These results have been reinforced by a further more extensive validation study which involved over 750 patients from 20 centers of 11 countries [9].

As the PDCS is a recently developed instrument, some of its potential scopes have not yet been studied. To the best knowledge of the authors, the PDCS has not been used in interventional studies, and consequently, there are no data available demonstrating its capability to detect clinical change. Therefore, we aimed to investigate the responsiveness of the PDCS to an acute levodopa challenge.

## 2. Materials and Methods

The study protocol was similar to the procedure utilized by Merello et al. to analyze the performance of the MDS-UPDRS motor section assessing the response to an acute levodopa challenge [10]. A consecutive series of patients with parkinsonism, undergoing an acute levodopa challenge at the Department of Neurology, University of Pécs, Hungary, between 2017 and 2018 were enrolled in this prospective study. The study protocol was approved by the Regional and Institutional Ethical Committee (3617.316-24987/KK41). In de novo or early-phase patients, the acute dopaminergic challenge was used for helping the differential diagnosis of parkinsonian syndromes, while in advanced PD cases it is used for evaluating the feasibility of patients for deep brain stimulation. If an adverse event interfering with the outcome (e.g., nausea, vomiting, and hypotension) occurred during the test, the patient was excluded. To minimize these events, patients were pretreated with domperidone (30 mg/day) 12–72 hours before and on-demand again 1 hour before the administration of levodopa.

In the case of those patients who were on antiparkinsonian medication, any form of levodopa was discontinued at least 12 hours prior (usually an overnight withdrawal) to achieve an OFF state. Long-acting dopamine agonists (e.g., pramipexole, ropinirole, and rotigotine) and monoamine oxidase-B inhibitors (e.g., rasagiline and selegiline) were withdrawn at least for 48 hours.

All examinations were performed by two highly experienced nurse practitioners. First, we assessed the motor sections of the MDS-UPDRS and the PDCS in the morning between 8 and 9 a.m. corresponding to an OFF state. Subsequently, we rated these instruments in ON state usually 60–90 minutes after the administration of 200–400 mg immediate-release formulation of levodopa/benserazide pills (Madopar® Dispersible, Roche). Those patients, who had chronically been treated previously with antiparkinsonian medications, were asked to decide whether the achieved ON state corresponds to their best ON. If not, further 50–100 mg levodopa was administered and this dose was repeated until the best ON state was achieved.

In addition to the MDS-UPDRS Part III and the PDCS, further neurological and neuropsychological examinations were performed for the better characterization of the study population. The severity of PD-related symptoms was globally assessed by the Hungarian validated versions of the other parts of the MDS-UPDRS [11], the HYS, and the CISI-PD. To assess nonmotor symptoms globally, the validated Hungarian version of the NMSS [12] was also included. Besides, neurocognitive performance, apathy, anxiety, and depression were also assessed by the MoCA [13, 14], the Lille Apathy Rating Scale [15], the Parkinson Anxiety Scale [16], the Montgomery–Asberg Depression Rating Scale [17], and the Parkinson's Disease Sleep Scale 2nd version [18, 19]. The 39-item Parkinson's Disease Questionnaire [20] was used to measure the disease-specific health-related quality of life.

The IBM SPSS software package (version 24.0.2, IBM Inc., Armonk, NY, USA) was used for statistical calculations. To test normality, Shapiro–Wilk test was utilized. Because some data from the applied scales did not follow the normal distribution, median and 25–75 percentiles were also shown besides the mean ± standard deviation values.

For correlation, Spearman's rank correlation coefficients were calculated by analyzing the association between MDS-UPDRS and PDCS motor changes due to the acute levodopa challenge. For correlation coefficients, the values 0–0.29 were indicative of weak correlation, the values 0.30–0.59 were indicative of moderate association, and the values 0.60–1.00 were considered as high association [21].

There is no exact definition for the clinically relevant response to acute levodopa challenge available. A consensus report suggested that at least 20–30% improvement in UPDRS III is required depending on the aim of the testing [22]. In a more recent single-center study, Merello et al. suggested that a 24.5% improvement in the MDS-UPDRS Part III seems to be clinically relevant [10]. Therefore, we considered the acute levodopa challenge positive if at least 24.5% improvement was documented on the MDS-UPDRS Part III.

Subsequently, receiver operating characteristic (ROC) analysis was performed. Improvements in the PDCS motor score corresponding to 20%, 24.5%, and 30% improvements in the MDS-UPDRS Part III were identified. To calculate the best cutoff values with the most optimal sensitivity and specificity, the Youden method was used [23]. The statistical significance level was set at 0.01.

## 3. Results

A total of 100 consecutive patients (47 females), aged 66.0 ± 9.7 years, were enrolled. Of them, 20 patients had de novo or early phase of parkinsonism. The mean duration of disease was 4.7 ± 4.5 years. Almost half of the patients (49%) suffered from mild (HYS 1&2) parkinsonism, while 23 patients had moderate (HYS 3) and 28 patients had severe (HYS 4&5) stage. Baseline characteristics of the study population (e.g., demographic and disease-specific data) are presented in Table 1.

Levodopa test was positive in 83 cases, while negative results indicated other parkinsonian syndromes responsible for the symptoms in 17 patients (Table 2). Mean MDS-UPDRS and PDCS motor scores were 45.1 ± 15.3 and 13.7 ± 6.2 points in OFF and 33.2 ± 15.2 and 10.1 ± 6.5 points in ON state, respectively. According to these data, an average of 27.0 ± 20.1% (−11.9 points) and 28.7 ± 30.3% (−3.6 points) reduction of MDS-UPDRS and PDCS motor scores could be achieved by administering levodopa (Table 2). Changes in the motor scores of the MDS-UPDRS and the PDCS occurring during an acute levodopa challenge by disease type are shown in Table 3.

**Figure 1:**
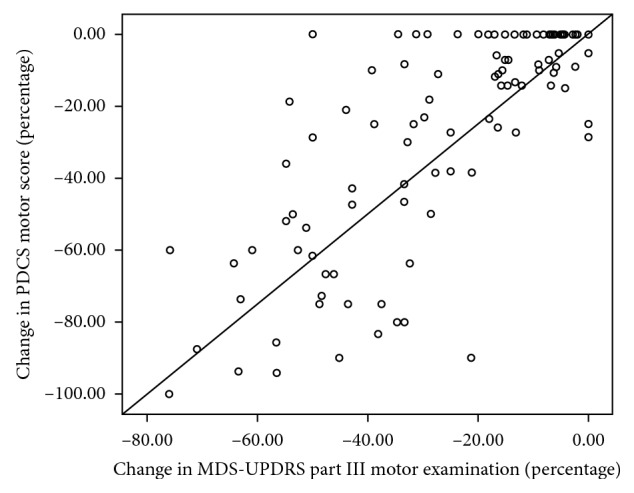
Correlation between the changes in motor scores of the MDS-UPDRS and the PDCS. Changes in the motor scores are presented in percentage.

High level of correlation (Spearman's rho = 0.726, *p* < 0.001) was found between changes in MDS-UPDRS and PDCS motor scores (Figure 1). The level of correlation varied according to the disease type from 0.465 (non-PD group) to 0.806 (tremor-dominant PD, Table 3).

**Figure 2:**
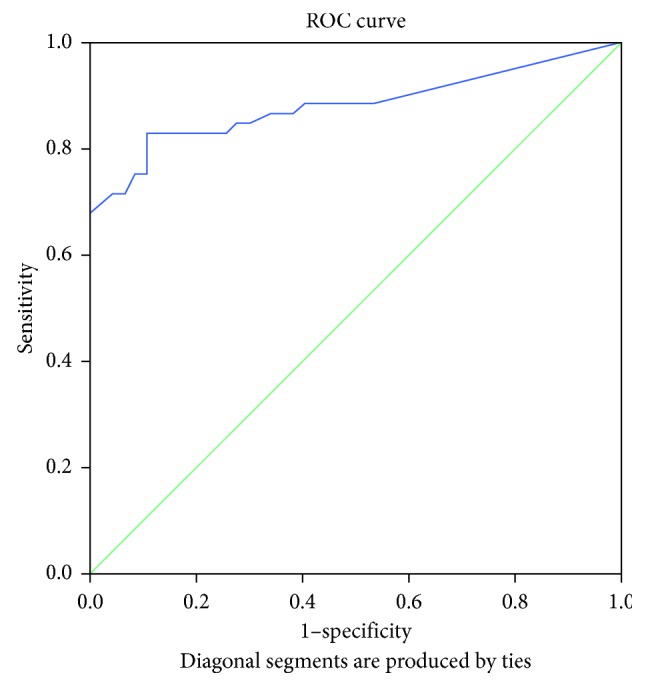
Receiver operating characteristic curve for illustrating the discriminating ability of the change in the PDCS motor score corresponding to a 20% change in the MDS-UPDRS Part III (motor examination).

**Figure 3:**
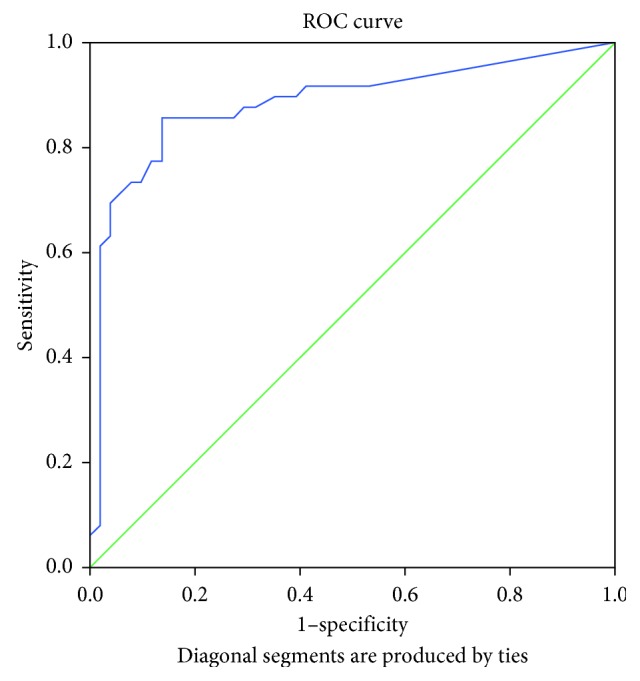
Receiver operating characteristic curve for illustrating the discriminating ability of the change in the PDCS motor score corresponding to a 24.5% change in the MDS-UPDRS Part III (motor examination).

**Figure 4:**
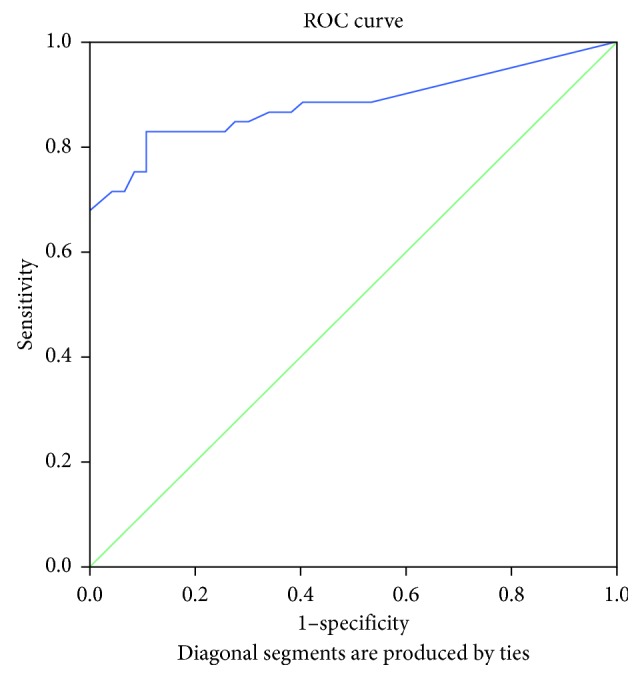
Receiver operating characteristic curve for illustrating the discriminating ability of the change in the PDCS motor score corresponding to a 30% change in the MDS-UPDRS Part III (motor examination).

The area under the ROC curve (AUC) for the change in PDCS motor score corresponding to the clinically relevant 20% improvement in the MDS-UPDRS Part III was 0.883 (*p* < 0.001; Figure 2). The area under the ROC curve for the improvement in PDCS motor score corresponding to the 24.5% change in the motor examination part of the MDS-UPDRS was 0.885 (*p* < 0.001; Figure 3), while the area under the ROC curve for the change in PDCS motor score corresponding to the 30% improvement in the MDS-UPDRS motor score was 0.883 (*p* < 0.001; Figure 4). The cutoff values for improvements in the PDCS motor scores, which indicate a clinically relevant response to acute levodopa challenge with the most optimal sensitivity and specificity and correspond to the 20%, 24.5%, and 30% changes in the MDS-UPDRS Part III, were 14.6%, 16.6%, and 18.5% (Table 4).

## 4. Discussion

To demonstrate the responsiveness of the motor domain of the PDCS to clinical change, we measured the improvement in both the MDS-UPDRS and the PDCS during acute levodopa challenge. Ideally, a clinical scale, such as the PDCS, should adequately detect the improvement in motor symptoms and differentiate the responders from the nonresponders.

We calculated the correlation between the changes in motor scores of the PDCS and the MDS-UPDRS, and we determined subsequently the minimum required improvement in the motor score of the PDCS corresponding to a clinically relevant improvement.

Internationally accepted diagnostic criteria of United Kingdom Parkinson's Disease Society Brain Bank for idiopathic Parkinson's disease include the responsiveness of motor signs of the disease to levodopa [24]. Therefore, an acute levodopa challenge, which is the standard way of testing this supportive prospective criterion, can help confirm or refute the clinical diagnosis of the disease.

Motor examination parts of the UPDRS and the MDS-UPDRS are the standard tools for measuring the improvement of motor symptoms developing due to the administration of a single dose of levodopa. At present, only a consensus-based definition of the clinically relevant response to acute levodopa challenge is available for clinical practice. According to this definition, if acute levodopa improves the motor score of a drug-naïve PD patient by at least 20% compared to baseline, the acute challenge can be considered as positive. However, a positive acute response to levodopa should be defined based on the aim of testing in treated patients [22]. A minimum threshold of 30% improvement in motor score compared to baseline was accepted as a clinically relevant change, indicating a positive chronic response to levodopa [22]. This empirically adopted 30% improvement has been confirmed to be a sensitive and specific threshold value predicating sustained long-term levodopa response by Merello et al. [25]. To conclude, current recommendations consider the 20% or 30% improvements in the UPDRS Part III to be clinically relevant. However, such threshold values are not available for the MDS-UPDRS. Although Merello et al. described that an approximately 24.5% improvement in the MDS-UPDRS Part III corresponds to an approximately 30% improvement in the UPDRS Part III [10], this finding has not yet been confirmed by other groups which make its generalizability uncertain. A major weakness of the previously established cutoff values may be that they can be highly sample dependent which considerably interferes with their applicability in clinical research. We, therefore, calculated the discriminating threshold values of PDCS motor score for both the 20%, 24.5%, and 30% improvements in the MDS-UPDRS Part III and, as a result, established a range between 14.6% and 18.5% improvements in the PDCS motor score which may be more applicable, compared to an exact threshold value, even for study populations differing from the present sample.

Fulfilling our expectations, we found a good and significant correlation between MDS-UPDRS Part III changes and PDCS motor score changes. We also demonstrated that PDCS can differentiate responders and nonresponders to levodopa. The cutoff values of 20%, 24.5%, and 30% in the motor examination part of the MDS-UPDRS for sustained levodopa response were equivalent to 14.6%, 16.6%, and 18.5% improvements in the PDCS motor score.

Merello et al. have demonstrated an excellent correlation between the motor examination parts of the UPDRS and the MDS-UPDRS both before (Pearson's *R* = 0.965) and after (Pearson's *R* = 0.968) acute levodopa challenge [10]. The correlation coefficient between the MDS-UPDRS Part III and the PDCS motor scores, which was found by a recent validation study using data of 776 patients (the findings of this study are awaiting for publication), was high (Spearman's *R* = 0.81) but not as strong as the correlation coefficient between the UPDRS and its successor version (MDS-UPDRS) found by Merello et al. We also found a good but not an excellent correlation between the changes in motor scores of the MDS-UPDRS and the PDCS during acute levodopa challenge. A possible reason for the lower correlation coefficient between these two scales (MDS-UPDRS and PDCS) may be the shorter number of items of the PDCS and the heterogeneous scoring of the PDCS motor items, which results from the weighting of the items based on clinical relevance of a particular symptom, in comparison with the motor parts of the UPDRS and the MDS-UPDRS having more items to correlate which are scored uniformly.

The strength of our study partly lies in the study population which also included patients with non-PD parkinsonism. Another strength of the present study may be that we used a range of improvement in the MDS-UPDRS motor scores indicating a clinically relevant response to levodopa for the identification of a range of variation in the PDCS motor scores demonstrating good levodopa response. The use of a range instead of an exact threshold value may provide the wider and more convenient applicability of our results because they are less dependent on the characteristics of the investigated patient population and they, therefore, should not be newly calculated for each study in which they are planned to be used.

To conclude, the PDCS seems to be adequately responsive to the acute levodopa challenge based on the good correlation between changes in the MDS-UPDRS and PDCS motor scores during the test. Any improvement in the PDCS motor score equal to or greater than 14.6% seems to demonstrate levodopa responsiveness. These results may be helpful for centers which plan to integrate the use of PDCS motor section into their protocols for performing acute levodopa challenge.

## Figures and Tables

**Table 1:** Baseline characteristics of the study population.

		Mean or count	Standard deviation or percentage	Median	25th percentile	75th percentile
Age (years)		66.0	9.7	66	60	74
Disease duration (years)		4.7	4.5	4	1	7
Levodopa duration (years)		4.7	4.3	4	1	7
Sex	Males	53	53.0%			
	Females	47	47.0%			
Handedness	Right	96	96.0%			
	Left	4	4.0%			
Dominant side	Right	33	37.5%			
	Left	55	62.5%			
BMI (kg/m^2^)		26.3	5.4	26.4	23.3	29.4
Education (years)		12.4	3.2	12	11	15
Disease type	Tremor-dominant PD	24	24.0%			
	Rigid-akinetic PD	35	35.0%			
	Mixed PD	24	24.0%			
	Non-PD (other parkinsonian syndromes)	17	17.0%			
De novo	No	75	75.0%			
	Yes	25	25.0%			
Hoehn and Yahr stage	Mild (1 and 2)	49	49.0%			
	Moderate (3)	23	23.0%			
	Severe (4 and 5)	28	28.0%			
MDS-UPDRS I. nM-EDL		14.2	7.4	15	7	19
MDS-UPDRS II. M-EDL		17.5	8.9	16	10	24
MDS-UPDRS III. ME		35.3	16.0	37	22	47
MDS-UPDRS IV. MC		5.0	4.0	4	2	7
MDS-UPDRS total score		71.9	28.9	72	48	93
PDCS motor score		13.4	6.3	13	10	18
PDCS nonmotor score		9.3	6.1	10	3	14
PDCS treatment-related complications score		5.5	3.9	4	2	9
PDCS disability score		2.4	1.9	2	1	4
PDCS total score		28.2	13.3	26	18	37
CISI total score		8.7	3.5	9	6	11
PDQ-39 summary index		29.1	16.5	30	16	41
MADRS total score		13.8	7.6	14	19	8
PAS total score		14.1	7.2	14	19	9
LARS total score		−21.1	10.9	−24	−16	−28
MoCA total score		22.4	5.5	24	27	18
PDSS-2 total score		19.9	11.8	19	28	10
NMSS I. Cardiovascular problems		3.9	4.3	2	8	0
NMSS II. Sleep problems		12.2	9.2	12	20	4
NMSS III. Mood problems		12.0	14.0	6	21	2
NMSS IV. Hallucinations		1.0	2.8	0	0	0
NMSS V. Memory problems		4.3	5.3	2	7	0
NMSS VI. Gastrointestinal problems		4.7	6.0	2	8	0
NMSS VII. Urinary problems		11.0	10.8	8	20	2
NMSS VIII. Sexual problems		0.8	2.3	0	0	0
NMSS IX. Miscellaneous		4.2	6.4	2	7	0
NMSS total score		54.1	39.6	49	82	20

BMI = body mass index; CISI = Clinical Impression of Severity Index; LARS = Lille Apathy Rating Scale; MADRS = Montgomery–Asberg Depression Rating Scale; MDS-UPDRS = Movement Disorder Society-sponsored Unified Parkinson's Disease Rating Scale; MDS-UPDRS I. nM-EDL = nonmotor experiences of daily living (Part I of MDS-UPDRS); MDS-UPDRS II. M-EDL = motor experiences of daily living (Part II of MDS-UPDRS); MDS-UPDRS III. ME = motor examination (Part III of MDS-UPDRS); MDS-UPDRS IV. MC = motor complication (Part IV of MDS-UPDRS); MoCA = Montreal Cognitive Assessment; NMSS = Non-Motor Symptoms Scale; PAS = Parkinson Anxiety Scale; PD = Parkinson's disease; PDCS = Parkinson's Disease Composite Scale; PDSS-2 = Parkinson's Disease Sleep Scale 2nd version; PDQ-39 = 39-item Parkinson's Disease Questionnaire.

**Table 2:** Average changes in MDS-UPDRS and PDCS motor scores during acute levodopa challenge.

	OFF state^*∗*^ (points)	ON state^*∗∗*^ (points)	Change (points)	Change (%)
MDS-UPDRS Part III	45.1 ± 15.3	33.2 ± 15.2	−11.9 ± 10.1	−27.0 ± 20.1
PDCS motor score	13.7 ± 6.2	10.1 ± 6.5	−3.6 ± 4.0	−28.7 ± 30.3

^*∗*^Any antiparkinsonian medication was discontinued at least 12 hours before the assessment. ^*∗∗*^60 minutes after a single dose of 200–400 mg immediate-release formulation of levodopa/benserazide or in the best ON state. Data are mean ± standard deviation. MDS-UPDRS = Movement Disorder Society-sponsored Unified Parkinson's Disease Rating Scale; PDCS = Parkinson's Disease Composite Scale.

**Table 3:** Average ON and OFF values and their changes in MDS-UPDRS and PDCS motor scores by disease type during levodopa challenge.

	Disease type			
	Tremor-dominant PD	Rigid-akinetic PD	Mixed PD	Non-PD (other parkinsonian syndromes)
MDS-UPDRS OFF (points)	45.3 ± 15.3	43.3 ± 16.5	48.3 ± 15.4	44.3 ± 13.1
MDS-UPDRS ON (points)	31.3 ± 13.5	29.6 ± 17.00	35.3 ± 13.9	40.5 ± 13.5
MDS-UPDRS change (points)	−14.0 ± 10.6	−13.7 ± 10.3	−13.1 ± 10.5	−3.8 ± 2.4
MDS-UPDRS change (%)	−29.7 ± 20.6	−33.5 ± 21.6	−26.9 ± 16.7	−9.9 ± 8.3
PDCS OFF (points)	12.0 ± 5.8	14.0 ± 6.8	14.2 ± 5.7	14.8 ± 6.0
PDCS ON (points)	8.0 ± 5.4	8.8 ± 7.0	11.3 ± 5.8	14.1 ± 6.0
PDCS change (points)	−4.0 ± 3.8	−5.2 ± 4.7	−2.9 ± 3.2	−0.7 ± 1.0
PDCS change (%)	−33.0 ± 28.0	−42.0 ± 34.7	−21.6 ± 24.5	−5.6 ± 8.2
Correlation (Spearman's rho) between changes in MDS-UPDRS (%) and PDCS (%)	0.806 (*p* < 0.001)	0.776 (*p* < 0.001)	0.685 (*p* < 0.001)	0.465 (*p* < 0.001)

Data are mean ± standard deviation. Assessments in OFF state were performed at least 12 hours after the discontinuation of any antiparkinsonian medication. Patients were reassessed 60 minutes after a single dose of 200–400 mg immediate-release formulation of levodopa/benserazide or in the best ON state. MDS-UPDRS = Movement Disorder Society-sponsored Unified Parkinson's Disease Rating Scale; PD = Parkinson's disease; PDCS = Parkinson's Disease Composite Scale.

**Table 4:** Corresponding changes in the motor scores of the Parkinson's Disease Composite Scale to the clinically relevant 20%, 24.5%, and 30% changes in the motor examination part of the MDS-UPDRS^*∗*^.

	PDCS improvement (%)	Sensitivity	Specificity	Youden's index	+LR	−LR	AUC	ROC *p* value
MDS-UPDRS 30% improvement	18.47	0.811	0.894	0.705	7.626	0.211	0.883	<0.001
MDS-UPDRS 24.5% improvement	16.59	0.857	0.863	0.720	6.245	0.166	0.885	<0.001
MDS-UPDRS 20% improvement	14.64	0.830	0.872	0.703	6.503	0.195	0.883	<0.001

The cutoff values were proposed as benchmarks based on [10, 22]. AUC = area under the curve; +LR = positive likelihood ratio; −LR = negative likelihood ratio; MDS-UPDRS = Movement Disorder Society-sponsored Unified Parkinson's Disease Rating Scale; PDCS = Parkinson's Disease Composite Scale; ROC = receiver operating characteristic.

## Data Availability

The data used to support the findings of this study have not been made available because the current ethical approval does not permit its deposition.

## Abbreviations

AUC: Area under the curve
CISI-PD: Clinical Impression of Severity Index for Parkinson's Disease
HYS: Hoehn and Yahr Scale
MDS-UPDRS: Movement Disorder Society-sponsored Unified Parkinson's Disease Rating Scale
MoCA: Montreal Cognitive Assessment
NMSS: Non-Motor Symptoms Scale
PD: Parkinson's disease
PDCS: Parkinson's Disease Composite Scale
ROC: Receiver operating characteristic.
